# Proteomic and genomic analysis of acid dentin lysate with focus on TGF-β signaling

**DOI:** 10.1038/s41598-021-89996-6

**Published:** 2021-06-10

**Authors:** Jila Nasirzade, Zahra Kargarpour, Goran Mitulović, Franz Josef Strauss, Layla Panahipour, Frank Schwarz, Reinhard Gruber

**Affiliations:** 1 Department of Oral Biology, Dental School, Medical University of Vienna, Sensengasse 2a, 1090 Vienna, Austria; 2 Clinical Department of Laboratory Medicine Proteomics Core Facility, Medical University Vienna, Vienna, Austria; 3 Clinic of Reconstructive Dentistry, Center of Dental Medicine, University of Zurich, Zurich, Switzerland; 4 Department of Conservative Dentistry, School of Dentistry, University of Chile, Santiago, Chile; 5 Department of Oral Surgery and Implantology, Johann Wolfgang Goethe-University, Frankfurt, Germany; 6 Department of Periodontology, School of Dental Medicine, University of Bern, Bern, Switzerland

**Keywords:** Preclinical research, Dental implants

## Abstract

Particulate autologous tooth roots are increasingly used for alveolar bone augmentation; however, the proteomic profile of acid dentin lysate and the respective cellular response have not been investigated. Here we show that TGF-β1 is among the 226 proteins of acid dentin lysate (ADL) prepared from porcine teeth. RNA sequencing identified 231 strongly regulated genes when gingival fibroblasts were exposed to ADL. Out of these genes, about one third required activation of the TGF-β receptor type I kinase including interleukin 11 (IL11) and NADPH oxidase 4 (NOX4). Reverse transcription-quantitative polymerase chain reaction and immunoassay confirmed the TGF-β-dependent expression of IL11 and NOX4. The activation of canonical TGF-β signaling by ADL was further confirmed by the phosphorylation of Smad3 and translocation of Smad2/3, using Western blot and immunofluorescence staining, respectively. Finally, we showed that TGF-β activity released from dentin by acid lysis adsorbs to titanium and collagen membranes. These findings suggest that dentin particles are a rich source of TGF-β causing a major response of gingival fibroblasts.

## Introduction

Autogenous tooth roots have brought a new approach to the field of oral bone augmentation^1^. Tooth roots were introduced due to the structural similarity between dentin and alveolar bone. Clinically, autogenous tooth roots have shown promising results in bone augmentation^2^ and prior to implant placement^3^ leading to similar outcomes compared to bone substitutes. Radiological analysis^4,5^, preclinical studies^6–8^ and case reports^9^ support the application of the autogenous tooth roots as a graft material. Moreover, as dentin is free of costs and easily accessible, the use of autogenous tooth roots has received increasing attention in alveolar bone augmentations. The rationale of this clinical approach rests on the similarities between dentin and bone in terms of biological and structural features^10,11^. Similarly to bone^12,13^, the proteomic pattern of dentin treated with chelating agent was recently reported^14^ nevertheless the respective cellular responses remain to be clarified.

Dentin, analogous to bone, is a rich source of growth factors including transforming growth factor-β (TGF-β)^14,15^. For example, TGF-β accumulates in the irritant harvested during endodontic root canal treatment^16,17^. Acidic digestion of bone and likely of dentin leads to the release of TGF-β, which in turn might support the cellular mechanisms leading to the consolidation of bone grafts^18^. There is indirect evidence that TGF-β released by acidic lysis during bone resorption controls migration of mesenchymal cells that later become bone-forming osteoblasts^19^. Osteoclastic bone resorption and the concomitant release of TGF-β and other growth factors presumably support the early stages of graft consolidation, even though the evidence is scarce^20^. Whereas the biological activity of TGF-β released from bone by acid lysis has been recently reported^13^, there are no studies about the biological activity of acid dentin lysate. There is thus a demand to identify TGF-β and other growth factors released from dentin upon acid lysis and to study the respective cellular responses.

Previous work on proteomic profiling of dentin was based on EDTA, an chelating agent^14^. We have chosen pH neutralized acid dentin lysate for proteomic profiling. We also report here the gene expression changes of gingival fibroblasts exposed to ADL, similar to what we have done with acid bone lysate^13^ and platelet-rich fibrin lysate^21^. Both studies identified interleukin 11 (IL11) and NADPH oxidase 4 (NOX4) to be among the most strongly regulated genes in gingival fibroblast cells requiring TGF-β receptor type I kinase^13,21^, and showed the phosphorylation and nuclear translocation of Smad3 and Smad2/3, respectively^22^. IL11 and NOX4 are not simply target genes as they are critically involved in mediating downstream TGF-β effects in cardiovascular and liver fibrosis^23,24^ and systemic sclerosis^25^. Here we determined the TGF-β activity of acid dentin lysate by using the established bioassay strategy that we followed for the analysis of acid bone lysate^13^.

In the present investigation we report that (i) TGF-β1 is among the proteomic profile of acid dentin lysate; (ii) which provokes a robust activation of genes including IL11 and NOX4 via the canonical TGF-β signaling in gingival fibroblasts; (iii) which is paralleled by the phosphorylation of Smad3 and the nuclear translocation of Smad2/3.

## Results

### Proteomics analysis of acid dentin lysate (ADL)

To determine the proteomic signature of acid dentin lysate (ADL) nano HPLC separation and mass spectrometry analysis of tryptically digested proteins were performed. Overall, 342 and 226 proteins were identified that show at least two and three detectable peptides, respectively (Supplementary Table 1). Among the proteins with three detectable peptides was TGF-β1, apart from the other growth factors IGF1, IGF2, PDGFD and CTGF and a series of binding proteins LTBP3, IGFBP2, and IGFBP5. The profile also consists of extracellular matrix proteins such as COL1A1, COL1A2, COL6A3, COL11A2 and COL12A1, usually being linked to small leucine-rich repeat proteoglycan that were also detected (DCN, BGN and LUM). The protein profile further revealed FN1, VIM, POST, OGN, all five porcine protegrins NPG1 to NPG5, MMP20 also known as enamel metalloproteinase or enamelysin, and ENAM, an enamel matrix protein. Moreover, keratins, histones, and ribosomal proteins were identified (Supplementary Fig. 2).

STRING analysis of ADL proteins resulted in 142 nodes with an average node degree of 12.2 and PPI enrichment p-value of less than 1.0e^−16^ (Fig. 1). Using kmean clustering method, 4 main clusters were found through STRING analysis, which include clusters of keratins, of histones and ribosomal proteins, of collagens and other members of the extracellular matrix, and of growth factors, chemokines and proteases, findings supported by the REVIGO analysis (Supplement Table 1).

**Figure﻿﻿ 1:**
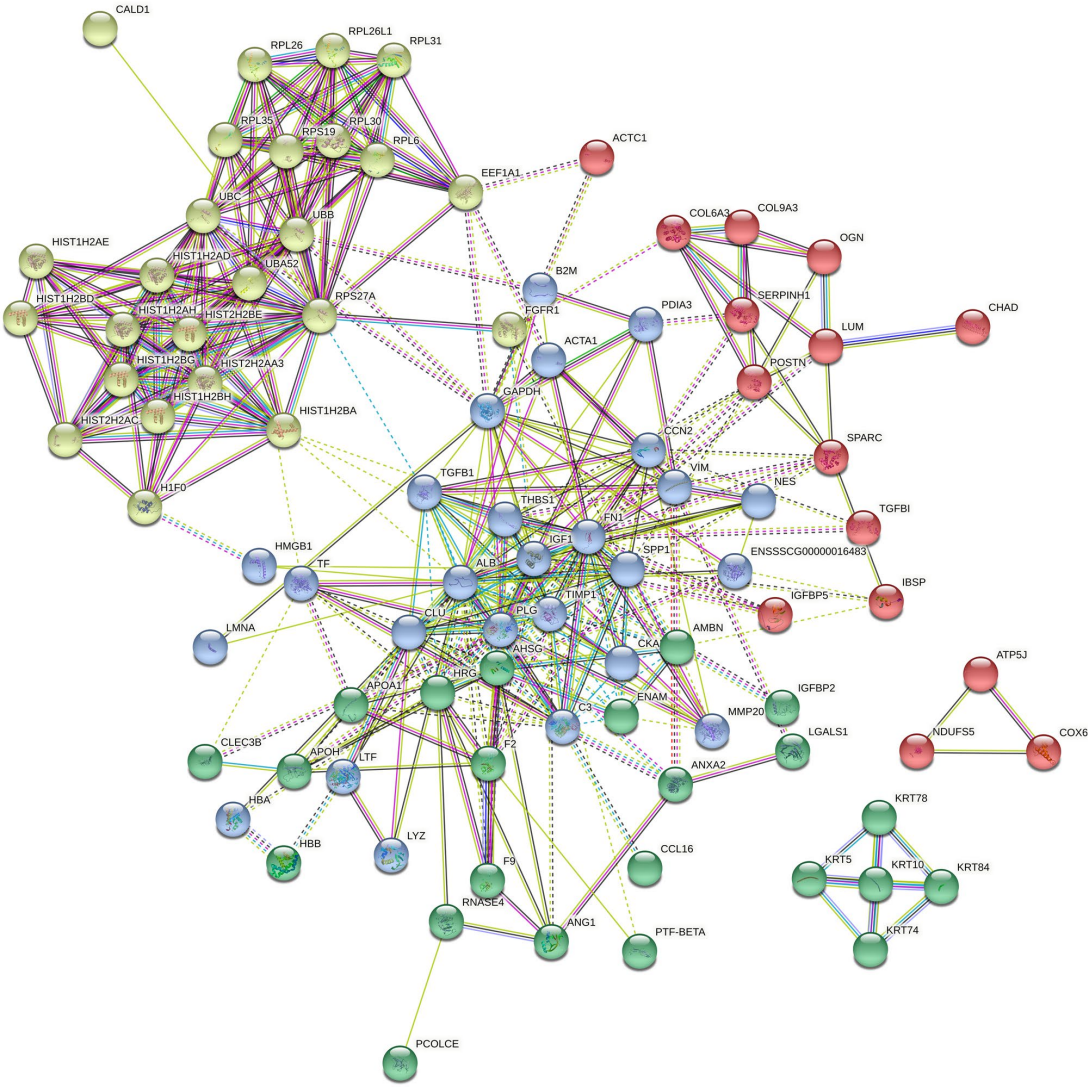
Protein clusters of acid dentin lysate (ADL). The STRING network shows proteins with at least three detectable peptides. Different line colors represent the types of evidence for the association between proteins. Based on kmean clustering method, network is categorized into four groups. There is a clear clustering of the histones and ribosomal proteins shown as yellow bubbles, and of five keratins shown as green bubbles. Not so sharp is the clustering of the collagens and other members of the extracellular matrix, some growth factors, chemokines and proteases. Network of interactions were depicted using String database (https://string-db.org).

### Viability of primary gingival fibroblasts exposed to ADL

**Figure 2:**
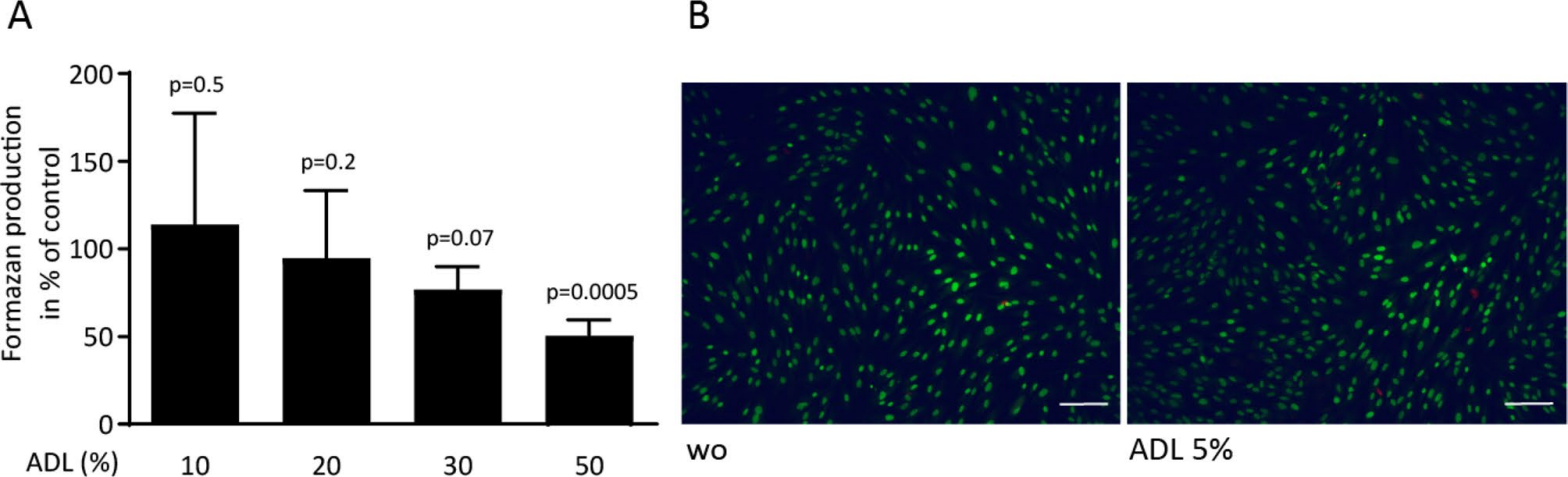
Viability of primary gingival fibroblasts exposed to ADL. Cell viability of primary gingival fibroblasts upon exposure to ADL was tested by (A) MTT assay and (B) Live-Dead staining. Stimulation with ADL at 5% is highly biocompatible with gingival fibroblasts. Live-Dead staining was performed with viable cells appearing in green and dead cells in red. “wo” represents unstimulated control. N = 3–5. Data represent the mean ± SD relative to the control. Statistic was based on multiple comparison on Scale bars indicate 200 µm.

To rule out any toxicity induced by ADL, cell viability tests with primary gingival fibroblast were performed. The MTT assay revealed that a concentration of 50% ADL was necessary to significantly decrease the production of formazan crystals (Fig. 2A). Live-Dead staining confirmed the lack of cytotoxicity with ADL at 5% (Fig. 2B). Together, these results indicate that stimulation of gingival fibroblasts with ADL up to 10% is feasible.

### RNA sequencing of gingival fibroblasts exposed to ADL

**Figure 3:**
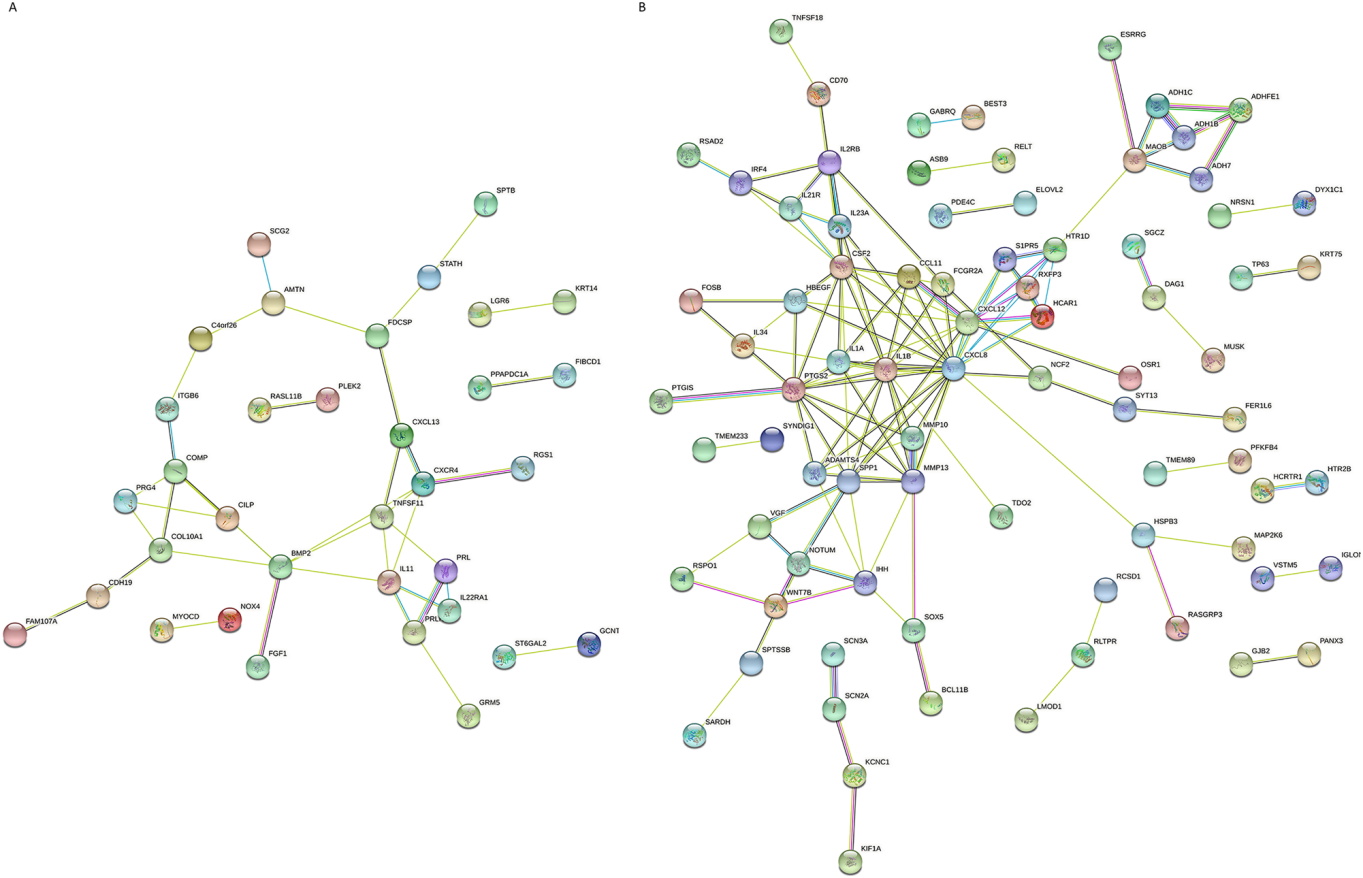
ADL induced gene expression in fibroblasts by RNAseq. STRING analysis of genes regulated by ADL which are (**A**) dependent and (**B**) independent of the TGF‐β RI kinase inhibitor. The number of unconnected bubbles, excluded from the figure is 43 and 72 in (**A**) and (**B**), respectively. (**A**) We noticed the presence of IL11, NOX4 and PRG4, which were already identified in previous studies to be regulated in a SB431542-dependent way. (**B**) Obvious are the expression of the inflammatory cytokines and chemokines, along with some of the proteases indicating an SB431542-independent proinflammatory cell response. Network of interactions were depicted using String database (https://string-db.org).

To identify the most strongly regulated genes by 5% ADL in gingival fibroblasts, an RNA sequencing approach was conducted. RNA sequencing revealed 231 genes among which 156 and 75 were at least 3log^2^-fold up- and downregulated by ADL, respectively. Among all ADL-regulated genes, 30%, that is 77 genes were blocked by the inhibitor for the TGF‐β RI kinase, SB431542, including IL11 and NOX4. We further identified 26 genes being downregulated by SB431542 but independently of ADL (Supplementary Table 2, Venny diagram available as Supplementary Fig. 3). STRING analysis of the genes regulated by ADL, dependent and independent of SB431542 resulted in 77 nodes with an average node degree of 1.01 and PPI enrichment p-value of less than 1.25^–8^ for ADL-regulated genes (Fig. 3A), and 153 nodes with an average node degree of 1.25 and PPI enrichment p-value of less than 1.01^–14^ for genes regulated by ADL depending on SB431542 (Fig. 3B).

GO enrichment analysis was performed on genes regulated by ADL in the presence or absence of SB431542, compared to untreated cells. For example, GO-term analysis of the SB431542-dependent (BMP2, CXCL13, CXCR4, IL11, IL22RA1, PRL, PRLR, TNFSF11) and SB431542-independent (CCL11, CD70, CSF2, CXCL12, CXCL8 (IL8), IL1A, IL1B, IL21R, IL23A, IL2RB, RELT, TNFSF18) genes, showed enrichment for hsa04060 cytokine-cytokine receptor interaction. REVIGO analysis was performed for ADL-regulated genes independent and dependent of SB431542 (Supplementary Table 2). Taken together, there is an enrichment of cytokines and chemokines that are regulated by ADL, suggesting that TGF-β1 and other ADL-derived molecules change the autocrine/paracrine behavior of gingival fibroblasts in vitro.

### ADL activates TGF-β signaling in human fibroblasts

**Figure 4:**
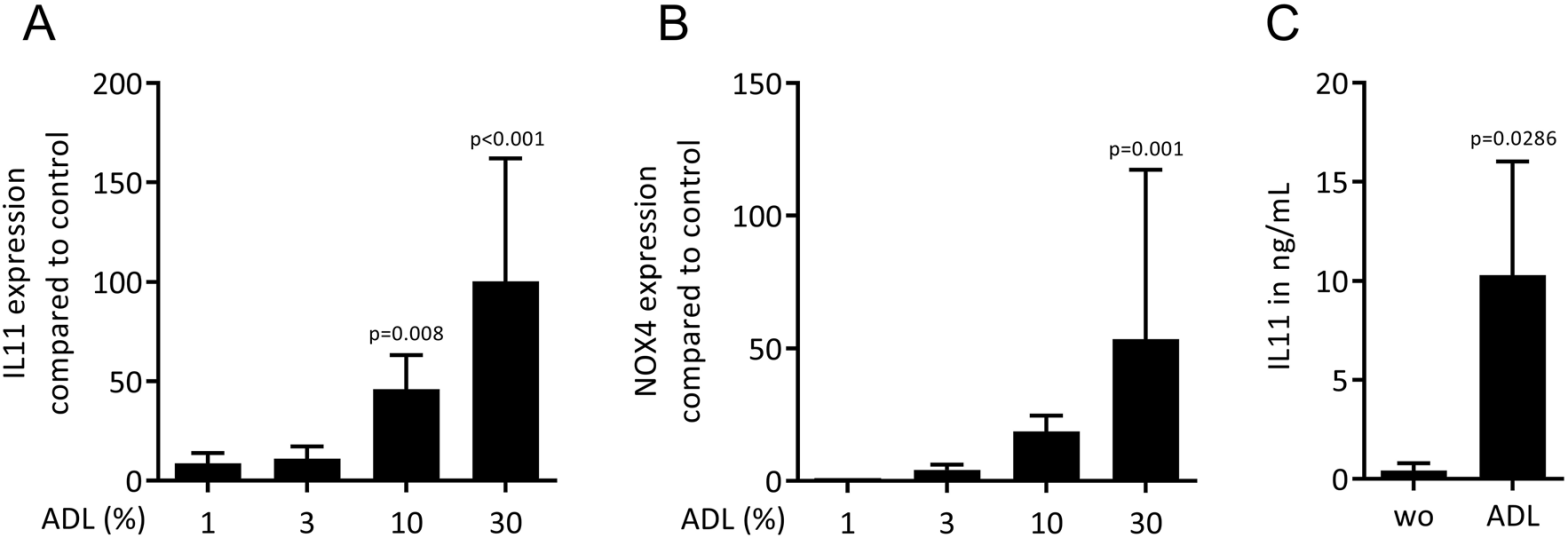
The effect of ADL on IL11 and NOX4 expression is dose-dependent. Gingival fibroblasts were incubated with various concentrations of ADL. Reverse transcription PCR analysis for (**A**) IL11 and (**B**) NOX4. Statistic was based on multiple comparison one-way ANOVA. (**C**) Quantification of IL11 in the supernatant by immunoassay. N = 3–4. Data are presented as mean ± SD. Statistic was based on two-tailed Mann–Whitney test.

**Figure 5:**
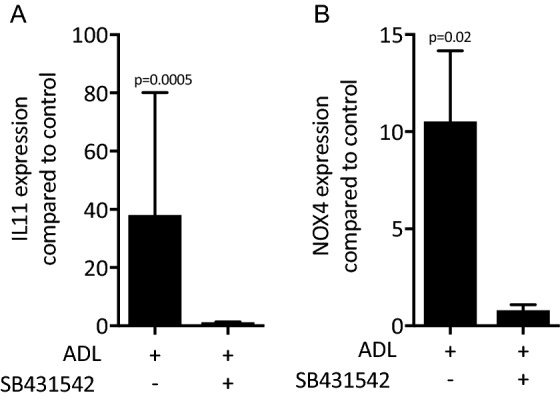
ADL changes the expression of IL11 and NOX4 via TGF-β receptor 1 kinase signaling. Addition of TGF-β receptor 1 kinase antagonist SB431542 to ADL blocks the expression of (**A**) IL11 and (**B**) NOX4. N = 3–5. Data represent the mean ± SD. Statistic was based on two-tailed Mann–Whitney test.

**Figure 6:**
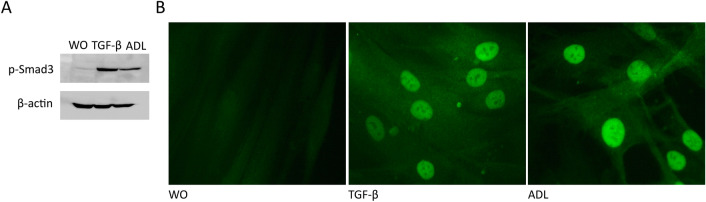
ADL induces phosphorylation of Smad3 and Smad2/3 signaling in primary gingival fibroblasts. Incubation of gingival fibroblasts with ADL caused (**A**) an increased phosphorylation of smad3 in Western blot. Representative immunofluorescence confirmed (**B**) the translocation of Smad2/3 into the nucleus upon stimulation with ADL. “WO” represents unstimulated control. Treatment with 10 ng/mL of TGF-β was used as a positive control. Scale bars indicate 50 µm.

To further confirm the TGF-β-dependent changes observed with the RNAseq approach, traditional RT-PCR was performed. In line with our previous findings using acid bone lysate^13^, ADL caused a dose-dependent increase of IL11 and NOX4 reaching the level of significance at around 10% ADL (Fig. 4A, B). That was confirmed at the protein level by an IL11 immunoassay (Fig. 4C). ADL from human teeth also increased IL11 expression in gingival fibroblasts (unpublished observation). The inhibitor for the TGF‐β RI kinase, SB431542 blocked the increased expression of IL11 and NOX4 in the presence of ADL (Fig. 5A, B). Activation of TGF‐β signaling was validated by Western blot and immunofluorescence showing an increased phosphorylation of smad3 (Fig. 6A) and a translocation of smad2/3 to the nucleus (Fig. 6B), respectively. Overall, these findings indicate a clear activation of TGF-β signaling pathway by ADL at different molecular levels.

### ADL-derived TGF-β activity binds to titanium surface and collagen membrane

**Figure 7:**
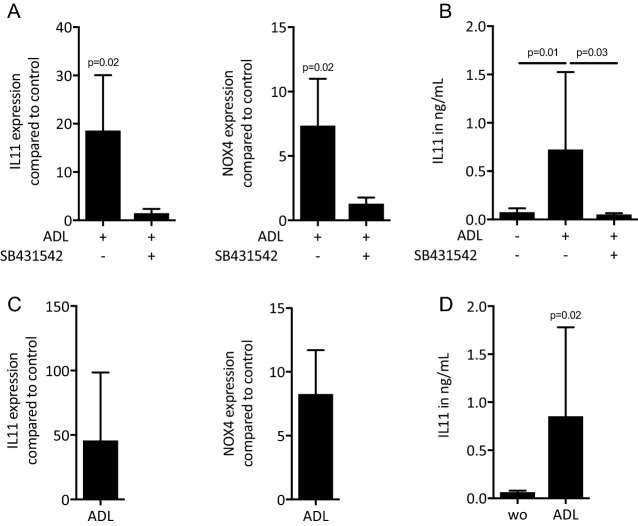
ADL-derived TGF-β activity absorbs to titanium surface and collagen membrane. Titanium discs were soaked in ADL for 1 h followed by three vigorous washes with phosphate buffered saline. Next, gingival fibroblasts were seeded onto the ADL coated titanium discs overnight with and without the inhibitor for the TGF-β RI kinase SB431542. Reverse transcription PCR analysis for (**A**) IL11 and NOX4 and (**B**) quantification of IL11 levels by immunoassay. Collagen membranes were treated with ADL for one h followed by three vigorous washes with buffered saline. Then, gingival fibroblasts were seeded onto the ADL-coated collagen membranes overnight. (**C**) Reverse transcription PCR analysis for IL11 and NOX4. (**D**) quantification of IL11 levels by immunoassay. N = 4–6. Data represent the mean ± SD. Statistic was based on two-tailed Mann-Whitney and Kruskal–Wallis test, for RT-PCR and immunosorbent assay, respectively.

We next evaluated binding of the TGF-β content of ADL to titanium surface and collagen membranes (Fig. 7). To this end, gingival fibroblasts were seeded on titanium discs that had been exposed to ADL and washed with phosphate buffer saline. Gingival fibroblasts showed a significant increase of IL11 and NOX4 gene expression along with a release of IL11 in the supernatant. These effects were inhibited by SB431542 (Fig. 7A, B). Then, gingival fibroblasts were seeded onto collagen membranes that had also been exposed to ADL. After strict washing of the membranes with phosphate buffer saline, similar to titanium, gingival fibroblasts substantially increased the expression of IL11 and NOX4 in line with the higher levels of IL11 in the supernatant, which were blocked by SB431542. Overall, these results indicate that TGF-β activity of ADL binds to titanium and collagen membranes (Fig. 7C, D).

### ADL reduces osteoblast differentiation in calvaria cells

**Figure 8:**
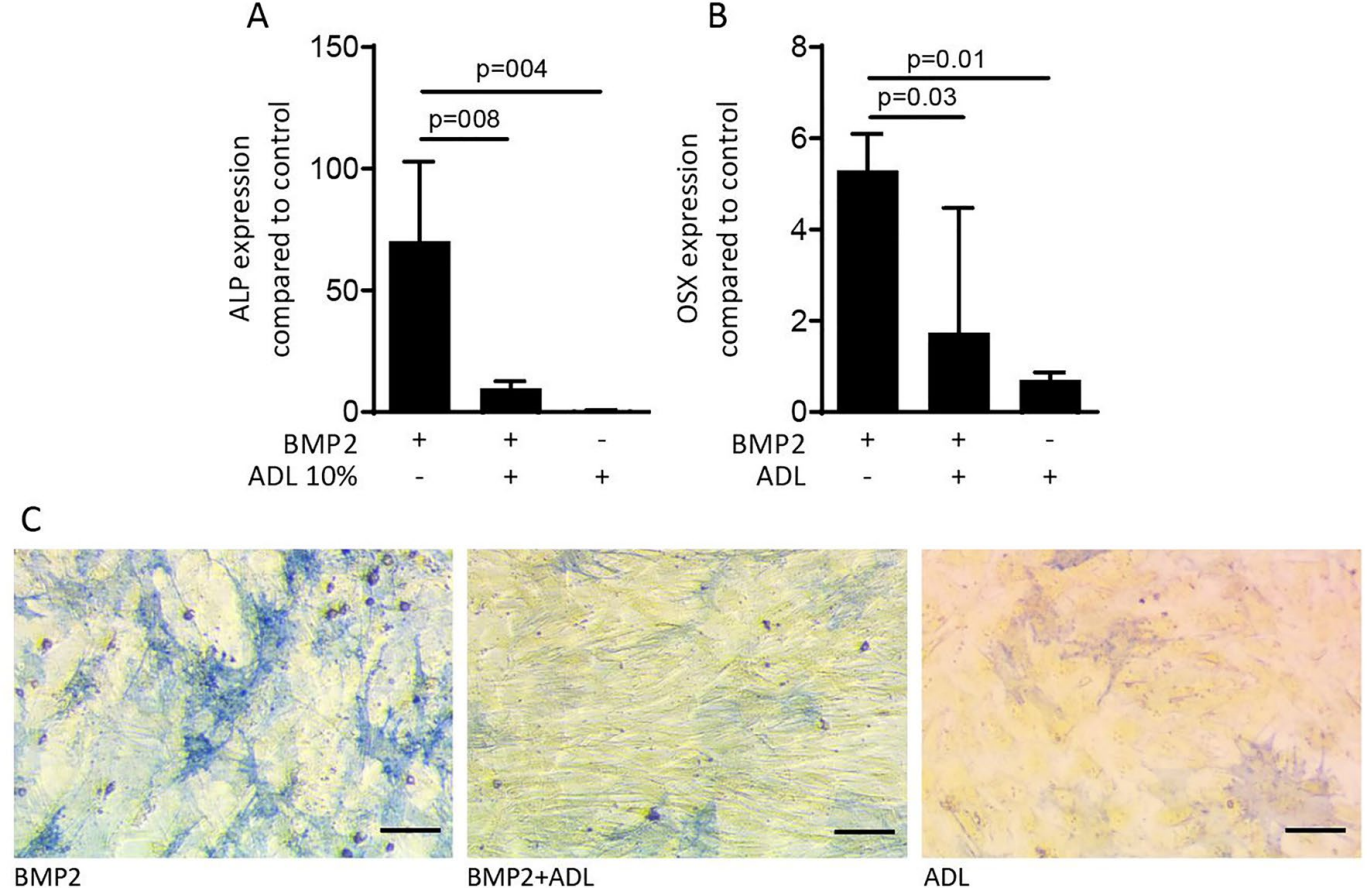
ADL decreases osteogenic differentiation of murine calvaria cells. Exposure of osteogenic calvaria cells to ADL caused a considerable decrease of the osteogenic marker genes (**A**) alkaline phosphatase (ALP) and (**B**) osterix (OSX). This was further confirmed by (**C**) the reduction of ALP staining induced by BMP2. N = 3–5. Data represent the mean ± SD, by Kruskal–Wallis test. Scale bars indicate 100 µm.

Finally, and given the known effect of TGF-β to suppress alkaline phosphatase in murine mesenchymal cells^26,27^, we tested the effect of ADL on osteogenic differentiation^13^. Exposure of calvaria cells from BALB/c mice of 2–3 day old, to ADL with and without BMP2 caused a significant reduction in the expression of alkaline phosphatase and osterix genes compared to BMP2 stimulated cells (Fig. 8A, B). This observation was further validated by histochemical staining of alkaline phosphatase showing a significant reduction of osteogenic differentiation in cells stimulated by ADL with and without BMP2 (Fig. 8C).

## Discussion

Accumulating evidence supports the clinical use of autogenous tooth roots for alveolar bone augmentation being free of costs and easily accessible^1,2,4–7,9^. Dentin undergoes creeping substitution during grafts consolidation, pointing towards the possibility that osteoclasts liberate TGF-β and other growth factors from the mineralized matrix which in turn serve as paracrine signals affecting cell behavior. If we accept the proposed acid treatment of dentin to simulate the paracrine microenvironment of osteoclast resorption, our paper provides insights into the various aspects relevant for graft consolidation; namely, to consider dentin as a source of TGF-β and other bioactive proteins that target cells including those of the mesenchymal lineage. Proteomic analysis of ADL identified 226 proteins including the classical growth factors TGF-β, IGF1 and IGF2 along with a series of other bioactive proteins such as chemokines. All these proteins can provoke a cellular response on target cells, including the gingival fibroblasts. This cellular response was examined by an RNAseq approach. We identified 231 at least ninefold regulated genes among which 30%, including IL11 and NOX4, required activation of the TGF‐β RI kinase. Moreover, consistent with our previous observations from acid bone lysate^13^, the TGF-β activity from ADL adsorbs to titanium and collagen membranes, suggesting a possible impact on guided bone regeneration^28^. Thus, the present research has identified dentin as a source of acid-soluble proteins and maybe other molecules that maintain their activity at low pH and provoke a complex response in gingival fibroblasts. This complex response depends to a great extent but not exclusively, on the activation of TGF-β signaling.

If we zoom into the qualitative proteomic signature of ADL, and in support of the previous proteomic analysis of dentin^14^ as well as pioneer research^15^, we could identify classic growth factors such as TGF-β1, IGF1 and IGF2, all of which are supposed to support bone remodeling and regeneration. For example, TGF-β released by the action of osteoclasts causes the immigration of mesenchymal progenitors which later become bone-forming osteoblasts^20^. IGF1 released by osteoclast from the bone matrix is also targeting mesenchymal cells^29^. Apart from the classical growth factors, other interesting proteins were detected; for instance, periostin promotes osteogenesis via supporting Wnt-signaling^30^, CTGF supports endochondral ossification^31^, and CXCL12/CXCR4 signaling is required for proper osteoblastogenesis and osteoclastogenesis^32^, with CXCL14 being the respective inhibitor^33^. Even though future studies should quantify the proteome, the qualitative proteome of ADL confirms the expected growth factors TGF-β and IGFs, as well as multiple other proteins, all of which could potentially target bone cells and thus modulate graft consolidation. Proteomics does not tell us, however, whether the proteins are biologically active after acid lysis. Considering the acid-stability of TGF-β and based on our previous data with acid bone lysate^13^ it was reasonable to suggest that TGF-β is an active components of ADL. In order to test this assumption, we performed an RNAseq analysis in fibroblasts exposed to ADL in the presence of the TGF-β receptor type I kinase inhibitor SB431542.

When gingival fibroblasts were exposed to ADL, the genetic signature dramatically changed. A total of 230 genes were strongly regulated under this condition and around one third of all genes required activation of the TGF‐β RI kinase. SB431542 modulated 104 genes including IL11 and NOX4. Apart from IL11 and NOX4, also BMP2, COL10A1, COMP, ESM1, PMEPA1, PRG4 were depending on SB431542, similar to acid bone lysate^13^. KEGG revealed that among the 74 genes, BMP2, CXCL13, CXCR4, IL11, IL22RA1, PRL, PRLR, TNFSF11 are involved in cytokine-cytokine receptor interaction; thus, the TGF‐β activity within ADL can change the autocrine/paracrine activity of the fibroblasts. For example, BMP2 is a potent growth factor required for bone regeneration^34^. CXCL13 produced by mesenchymal cells has a pathologic function^35^ but may also support bone regeneration^36^. CXCR4 builds an axis with CXCL12 for stem cell homing and bone regeneration^32^. TNFSF11/RANKL is an essential regulator of osteoclastogenesis^37^ and mammary gland development^38^, and PRL protects against osteoclastogenesis and bone loss in inflammatory arthritis^39^. IL11 accelerates bone regeneration^40^ and NOX4 deficient mice have increased bone density in the tibiae^41^. However, IL11^23,24^ and NOX4^25^ also mediate the effects of TGF‐β on fibrosis under pathological conditions. Taken together, the SB431542-dependent genes in fibroblasts are associated with bone regeneration and fibrosis.

There are a series of other genes strongly regulated by ADL but independent of SB431542. Among those genes, are the inflammatory cytokines and chemokines IL1A, IL1B, and CXCL8 as well as the other inflammatory mediators. For example, the IL23-IL17 axis^42^, CD27-CD70 axis^43^ (TNFSF7), CSF2 (GM-CSF)^44^, and TNFSF18^45^ are involved in the development of arthritis^46^ and RELT (TNFRSF19L) may regulate inflammation as well^47^. These genes are all upregulated and considered as “pro-inflammatory”. Even though the extracted teeth were mechanically cleaned with a tooth brush and dentifrice, there might be some dental plaque remnants with bacterial endotoxins that are capable of eliciting an inflammatory response^48,49^. In support of these observations, cleaning of extracted teeth reduced but not fully abolished the inflammatory response of macrophages to ADL^49^. Thus, the inflammatory response of the fibroblasts to ADL might be linked to the remaining dental plaque and not necessarily to some pro-inflammatory mediators originating from dentin.

Also independent of SB431542, ADL downregulated genes linked to tyrosine metabolism e.g., ADH1B, ADH1C, ADH7, MAOB. There is a link between TGF‐β and the expression of ADH1; TGF-β downregulates the expression of the alcohol metabolizing enzyme ADH1 via canonical TGF-β signaling^50^, whereas in the present study this change of gene expression was not affected by SB431542. Interestingly is the decreased monoamine oxidase B (MAOB) expression that can regulate bone turnover^51,52^. ADL further reduces sodium and potassium voltage-gated channels (SCN2A, SCN3A, and KCNC1) which are linked to the electrical stimulation on mesenchymal cells^53^. The potent bone activators WNT7B^54^ and IHH^55^ were increasingly expressed by ADL. Overall, there is indirect evidence that ADL might change the autocrine/paracrine function of fibroblasts and by this, modulate local bone regeneration. More research is necessary to identify the TGF‐β-independent pathway being activated by ADL in fibroblasts.

The presence of TGF-β in ADL supports earlier studies where a gradually release of TGF-β occurred upon endodontic treatment of tooth roots by acid^16,17^. Accordingly, our former work on acid bone lysate revealed the presence of active TGF-β^13^, which confirms the similarities between tooth and bone. In line with these findings, bone-conditioned medium (BCM) is enriched in TGF-β^56^. Consistent with the experimental approach of the aforementioned studies, we examined the TGF-β signaling pathway in human gingival fibroblasts at different molecular and cellular levels. The robust expression changes in the TGF-β target genes elicited by ADL are in accordance with previous results with BCM^56^ and ABL^13^, all showing an increase of IL11 and NOX4 expression. These changes were further confirmed in the present study by an increase of IL11 at the protein level^13^. Moreover, these changes were dependent on TGF-β receptor 1 kinase, since SB431542 suppressed all ADL effects, similar to what has been observed with ABL^13^. Based on previous studies showing that recombinant TGF-β induces phosphorylation and localization of Smad2/3^57^, we now show the phosphorylation and intracellular nuclear translocation of Smad2/3 in the presence of ADL. It should, however, not be ruled out that the activation of the canonical signaling pathway by ADL is strictly required for the expression of IL11, NOX4 and other SB431542-dependent genes.

On the search for other bioassays to show a TGF-β activity, we observed a strong suppression of osteogenic differentiation by ADL, indicated by alkaline phosphatase and osterix. Although this decrease in osteogenic differentiation is a hallmark of the TGF-β activity in vitro^13,26,27^, it should not be interpreted as suppression of bone formation^58^ since TGF-β has biphasic effects on osteoblastogenesis and osteoclastogenesis, depending on its concentration, time point, and duration of exposure of the target cells. Considering that TGF-β is not only critically involved in bone formation under physiologic^20^ but also in heterotopic ossification in mice^59^ our data are an indirect support for a possible beneficial effect of transplanted autogenous tooth roots on bone regeneration. Nevertheless, the lowering in osteogenic differentiation is a possible sign for the differentiation switch towards a fibroblastic phenotype that is linked to fibrosis, supporting the critical role of IL11 and NOX4 as mediators of this process^23–25^. Even though it is very unlikely that TGF-β released by osteoclasts from mineralized grafts forces the formation of a fibrous tissue, understanding why mesenchymal cells become osteoblasts rather than fibroblasts is a relevant future research question.

The clinical relevance of our findings remains a matter of speculation. Assuming that autogenous tooth roots are resorbed by osteoclasts causing the release of TGF-β and therefore the migration of pro-osteogenic mesenchymal cells^20,60^, autogenous tooth roots appear to be more than simple osteoconductive space holders. In fact, they are a source of TGF-β and a myriad of other growth factors and bioactive proteins that might modulate the local cellular events, culminating in the consolidation of the graft. Under this premise, the clinical relevance of our research is further supported by the observation that the TGF-β activity of ADL can adsorb to titanium surfaces and collagen membranes, suggesting an impact on GBR procedures. However, care should be taken not to overinterpret the findings of a cell culture-based in vitro study that may, if at all, only marginally represent the complex in vivo situation of creeping substitution and its impact on graft consolidation.

The present study has even more limitations and results should be reasonably interpreted. For example, it remains unclear if the ADL-derived growth factors released by the osteoclasts in vivo would reach the biomaterial surface in the presence of a blood clot. Fibrin^61^, vitronectin and fibronectin^62^ can adsorb TGF-β and work as a scaffold for the growth factors released from the dentin and other sources applied in a clinical setting. For example, platelet-rich fibrin^63^, demineralized bone grafts^64^ and even certain collagen-based biomaterials^65^ release a TGF-β activity. To come closer to an answer, we have recently evaluated the impact of ADL bound to a collagen membrane on in vivo bone formation in a rat calvaria defect model. Under these conditions, however, ADL failed to modulate bone regeneration^66^. These data should not be interpreted as failure as ADL is not a synonym for dentin being clinically used for bone augmentation. Our research is an attempt to understand the complexity of dentin as a bone graft and to show the complex cellular response of one out of many possible target cells, the fibroblast. Under this premise, we have unraveled the proteomic profile of ADL indicating TGF-β as one major signaling pathway accounting for the activation of one-third of genes expressed in gingival fibroblasts.

## Methods

### Acid dentin lysate (ADL)

Teeth were extracted from adult pigs 6 h after sacrification (Fleischerei Leopold Hödl, Vienna, Austria). Pigs were sacrificed not for the purpose of our experiments. Extracted teeth were cleaned from periodontal ligaments and soft tissue attachment with a surgical blade (Swann-Morton, Sheffield, United Kingdom) and then enamel was removed by using a manual grinding and polishing device (Metaserv 2000, Cleveland, Ohio). Subsequently, the pulp chamber was cleaned with a dental probe (Instrapac, Worksop, United Kingdom) and the teeth were grounded using a hammer. One gram of wet grounded dentin was incubated while being stirred overnight at room temperature with 10 ml of 0.1 N HCl (10% weight/volume). The resulting acid dentin lysate was then centrifuged, and the pH of the supernatant was neutralized. Following sterile filtration, the acid dentin lysate (ADL) were kept frozen at − 20 °C. The stocks were thawed immediately before each experiment.

### Cell culture

All experiments were performed in accordance with the relevant guidelines and regulations for human and animal research. Human gingiva was taken from extracted wisdom teeth of patients who had signed an informed consent. The harvesting procedure was approved by Ethics committee of Medical University of Vienna (EK NR 631/2007). Experiments were performed by three different strains of fibroblasts derived from the explants, passaged less than 10 times. Mouse calvaria-derived cells were isolated according to a standard protocol^67^. In brief, BALB/c mice aged 2–3 days were purchased from Animal Research Laboratories (Himberg, Austria). Organ donation from mice required an informal approval of the local veterinarian authorities but a formal approval by the Ethics Committee according to Austrian law. Euthanasia was performed by decapitation immediately prior to the organ donation. The calvaria was dissected and sequentially digested by 0.2% collagenase/dispase (Hoffmann-La Roche, Sigma, St. Louis, MO). The first two digests, each 20 min at 37 °C at continuous shaking, were discarded. Cells released by digests 3–5 were pooled and expanded. Human gingival fibroblasts and mouse calvaria cells were seeded in a number of 30,000 cells/cm^2^ incubated with ADL in serum-free medium, overnight. Additionally, machined titanium discs (Ti Gr. 4; Implacil De Bortoli, São Paulo, Brazil) and collagen membranes (Bio-gide, Geistlich Biomaterials, Wolhusen, Switzerland) were soaked in ADL for one hour at room temperature and washed with phosphate buffer saline for three times, as previously described^28^. Concentration of 5% for ADL was chosen from the dose-dependent stimulation of the cells by ADL. The inhibitor for the TGF‐β RI kinase, SB431542 (Calbiochem, Merck, Billerica, Massachusetts, USA) was applied at the concentration of 10 μM^21^.

### Mass spectrometry

The proteomic analysis was performed similar to what was reported for acid bone lysate^13^. Briefly, extracted proteins of ADL were precipitated by methanol/dichloromethane and digested using trypsin. Precipitated proteins were dissolved in 50 mM triethylammonium bicarbonate, and protein concentration was measured by DeNovix DS-11 Microvolume Spectrophotometer (Wilmington, USA). Proteins were digested overnight at 37 °C in a trypsin/protein ratio of 1:50. Peptides were separated on a C18 µPAC (µ-Pillar-Arrayed-Column, PharmaFluidics, Gent, Belgium) by a nano RSLC UltiMate3000 (ThermoScientific, Vienna, Austria) separation system and detected with a Q-Exactive Plus Biopharma mass spectrometer. An injection program defined by the user was applied for sample injection and extra injector and trap column wash. following every sample injection was two blank runs were conducted by injections of 2,2,2-trifluoroethanol for removal of any remaining samples in the injector or on the trap column and prevention of carryover in the separation system. All database searches were performed using the in-house Mascot 2.6 and the most recent version of the Sus scrofa SwissProt database. All search results were refined and researched using Scaffold 4.6.5 (Proteome Software, Portland, OR). Protein ontology and interactions were analyzed using String database (c). The mass spectrometry proteomics data have been put to the ProteomeXchange Consortium by the PRIDE partner repository^21^. Detailed description of the mass spectroscopy is provided in the supplementary file. To omit redundant GO terms, REVIGO analysis^68^ was performed considering a small allowed similarity of 0.5, whole UniProt as the database and SimRel as semantic similarity algorithm used. To conduct that, GO terms of each individual cluster together with its corresponding false discovery rate were uploaded in http://revigo.irb.hr. Gene function analysis was performed with the PANTHER classification system^69^. The online database STRING (https://string-db.org/) was applied to construct a PPI network of the genes followed by clustering of proteins.

### Cell viability

To evaluate the compatibility of the gingival fibroblasts with ADL, cells were exposed to defined concentrations of ADL, overnight. 0.5 mg/mL concentration of MTT (3-[4,5-dimethythiazol-2-yl]-2,5-diphenyltetrazolium bromide; Sigma) solution was added to each well of a microtiter plate (CytoOne, Sunnyvale, CA) for 2 h at 37 °C. The medium was discarded, and formazan crystals were solubilized in dimethyl sulfoxide. Optical density was read at 570 nm. Data were shown as percentage of optical density in the treatment groups normalized to untreated control group. In addition, Live-Dead staining (Enzo Life Sciences, Lausen, Switzerland) was performed to confirm the maintenance of gingival fibroblast cell viability in the presence of 5% ADL. The viability test methodology was previously reported^13^.

### RNA sequencing

Total RNA was extracted with the RNA Isolation Kit (Extractme, BLIRT S.A., Gdańsk, Poland). RNA quality was evaluated using the Agilent 2100 Bioanalyzer (Agilent Technologies, Santa Clara, CA, USA). Sequencing libraries were prepared at the Core Facility Genomics, Medical University of Vienna using the NEBNext Poly(A) mRNA Magnetic Isolation Module and the NEBNext Ultra II Directional RNA Library Prep Kit for Illumina according to manufacturer's protocols (New England Biolabs). Libraries were QC-checked on a Bioanalyzer 2100 (Agilent) using a High Sensitivity DNA Kit for correct insert size and quantified using Qubit dsDNA HS Assay (Invitrogen). Pooled libraries were sequenced on a NextSeq500 instrument (Illumina) in 1 × 75 bp single-end sequencing mode. Approximately 25 million reads were generated per sample. Reads in fastq format were aligned to the human reference genome version GRCh38 (www.ncbi.nlm.nih.gov/grc/human) with Gencode 29 annotations (www.gencodegenes.org/human/release_29.html) using STAR aligner^70^ version 2.6.1a in 2-pass mode. Reads per gene were counted by STAR, and differential gene expression was calculated using DESeq2^71^ version 1.22.2. DESeq2 results with a padj < 0.05 and a log2fc of >  = 3 or <  = − 3 (~ 9 × linear change). The resulting p-values were corrected for multiplicity by applying Benjamini–Hochberg adjustment to all *p*-values calculated for a time point with a false discovery rate (FDR) < 5%. Genes with an adjusted *p*-value < 0.05 were considered significant. REVIGO analysis, PANTHER classification, and STRING were performed.

### RT-PCR and immunoassay

Total RNA was extracted (ExtractMe) and exposed to reverse transcription (SensiFAST, Bioline Reagents Ltd., London, UK). RT-PCR was done according to the manufacturer’s instructions (LabQ master mix; LabConsulting, Vösendorf, Austria) on a CFX Connect PCR device (BioRad, Hercules, CA, USA). Primer sequences are given in Table 1, except for IL11, which was purchased from BioRad. Calculation of relative gene expression was based on delta delta CT method using a software (CFX Maestro, BioRad). Reactions were done in duplicates. The supernatant was evaluated for IL11 secretion by immunoassay according to the manufacture’s instructions (R&D Systems, Minneapolis, MN)^21^.

**Table 1:** Primer sequence.

	Sequence F	Sequence R
hGAPDH	aag cca cat cgc tca gac ac	gcc caa tac gac caa atc c
hßactin	cca acc gcg aga aga tga	cca gag gcg tac agg gat ag
hNOX4	ctc ctg gtt ctc ctg ctt gg	Gctgac gtt gca tgt ttcag
mGAPDH	act ttg gat tgt gga agg	gga tgc agg gat gat gtt ct
mßactin	cta agg cca acc gtg aaa ag	acc aga ggc ata cag gga ca
mALP	aac cca gac aca agc att cc	gag aca ttt tcc cgt tca cc
mOSX	ac atc cct ggc tgc ggc aa	ccg ggt gtg agt gcg cac at

### Western blot

Similar to what we have done recently^21^, proteins were extracted from the cells using lysis buffer which contained SDS and protease inhibitors (PhosSTOP with cOmplete; Sigma, St. Louis, MO). Proteins were then separated via SDS-PAGE and transferred onto PVDF membranes (Whatman, GE Healthcare, General Electric Company, Fairfield, CT). Membranes underwent blocking and were incubated with the first antibody (1:500; rabbit anti-pSmad3 Ser423/425, Abcam, ab52903, Cambridge, UK) and actin (Santa Cruz Biotechnology, Santa Cruz, CA) overnight. An antibody labelled with peroxidase (1:10,000; CS-7074, anti-rabbit IgG, Cell Signaling Technology) was used as secondary antibody. Peroxidase was visualized with Clarity Western ECL Substrate (Bio-Rad Laboratories, Inc., Hercules, CA, USA) and signals detected with the ChemiDoc imaging system (Bio‐Rad Laboratories, Inc. CA).

### Immunofluorescence

In line with our previous research^21^, immunofluorescent analysis was conducted on human gingival fibroblasts plated onto Millicell EZ slides (Merck KGaA, Darmstadt, Germany) stimulated with ADL 10% for 30 min. Cells were fixed using paraformaldehyde and blocked in 5% BSA and 0.3% Triton in PBS at room temperature for 1 h. Cells were next incubated with Smad2/3 antibody (1:800; D7G7 XP Rabbit mAb #8685, Cell Signaling Technology, MA, USA) overnight at 4 °C. Alexa Fluor 488 secondary antibody (1:1000; Anti-Rabbit, Cell Signaling) was added for 1 h. Cells were rinsed and put onto glass slides. Fluorescent images were taken at 4 × by a Zeiss Axiovert 200 M fluorescent microscope.

### Osteogenic differentiation

To investigate the effect of ADL on alkaline phosphatase (ALP) and osterix gene expression, calvaria cells were seeded in 24-well plates using Minimum Essential Medium Eagle-Alpha Modification (α MEM) supplemented with 10% fetal bovine serum and 1% antibiotics. Following 72 h of exposure to 300 ng/mL rhBMP2 (Prospec, Rehovot, Israel) with and without 10% ADL, gene expression analysis and histochemical staining of alkaline phosphatase were conducted. For ALP staining, cells were fixed as indicated and incubated with a substrate solution containing naphthol AS-TR phosphate and fast blue BB salt^72^. After rinsing with distilled water, cultures were photographed.

### Statistical analysis

Following the study design presented recently^21^, all experiments were done three to five times. Bars indicate the mean and standard deviation of the cumulative data from all experiments. Statistical analysis was done based on Mann–Whitney U test and multiple comparison one-way ANOVA. Analyses were performed using Prism 8 (GraphPad Software, La Jolla, CA). Significance was set at *p* < 0.05.

## Supplementary information

Supplementary information 1.

Supplementary information 2.

Supplementary Information 3.

## Data Availability

The datasets generated during and/or analyzed during the current study are available from the corresponding author on reasonable request.
