# Supplementary material for: Proteomic and genomic analysis of acid dentin lysate with focus on TGF-β signaling
Source: Sci Rep. 2021 Jun 10;11:12247. doi: 10.1038/s41598-021-89996-6 (PMC8192760; doi:10.1038/s41598-021-89996-6)
Supplement: Supplementary file 3 — Supplementary Information 3. [file 41598_2021_89996_MOESM3_ESM.docx]

**Supplementary Information**

**Proteomic and genomic analysis of acid dentin lysate with focus on TGF-β signaling**

Jila Nasirzade^1^, Zahra Kargarpour^1^, Goran Mitulović^2^, Franz Josef Strauss^1,3,4^, Layla Panahipour^1^, Frank Schwarz^5^, Reinhard Gruber^1,6^

^1^Department of Oral Biology, Medical University of Vienna, Vienna, Austria

^2^Clinical Department of Laboratory Medicine Proteomics Core Facility, Medical University Vienna, Austria.

^3^Clinic of Reconstructive Dentistry, Center of Dental Medicine, University of Zurich, Zürich, Switzerland

^4^Department of Conservative Dentistry, School of Dentistry, University of Chile, Santiago, Chile

^5^Department of Oral Surgery and Implantology, Johann Wolfgang Goethe-University Frankfurt, Germany

^6^Department of Periodontology, School of Dental Medicine, University of Bern, Bern, Switzerland

Reinhard Gruber; Department of Oral Biology, Dental School, Medical University of Vienna

Sensengasse 2a, 1090 Vienna, Austria; Phone: + 43 1 40070 2660, Email: reinhard.gruber@meduniwien.ac.at

**Supplementary files:**

Supplementary table 1: Tables related to proteomic data

Supplementary table 2: Tables related to RNA sequencing data

# Supplemental Methods

*Mass spectrometry*

Extracted proteins were precipitated using methanol/dichloromethane and digested with trypsin as described earlier (Fichtenbaum et al., 2016). Briefly, following acidic hydrolysis, proteins were dissolved in 50mM TEAB digestion buffer, pH 8.5 and were precipitated again using the modified Wessel-Fluegge method. Protein concentration was determined using the DeNovix DS-11 FX Spectrophotometer (Wilmington, USA) and were reduced using 5mM DTT (Dithiothreitol, Iodoaceticamide, Sigma-Aldrich, Vienna, Austria) for 30 minutes at 60°C, and alkylated for 30 minutes with 15mM IAA (Iodoaceticamide, Sigma-Aldrich, Vienna, Austria) in the dark. Finally, porcine trypsin (Promega, Vienna, Austria) was added in a ratio 1:50 (w/w). After 16 hours of incubation at 37°C, aliquots of 20µl were prepared and stored in 0.5ml protein low-bind vials (Eppendorf, Vienna, Austria) at -20°C until injection on next day. The nano HPLC Separation was performed using a nanoRSLC UltiMate 3000 HPLC system by Thermo Fisher. Mobile phases applied for sample loading, desalting, and separation were 0.01% aqueous heptafluorobutyric acid (HFBA) solution was used for sample loading applying a User Defined Program for sample injection. The loading mobile phase was delivered to the trap column at 30µl/min using the loading pump.

Mobile phases for peptide separation on the nano separation column were:

- A: 95% Acetonitrile (AcN), 5% Water, 0.1% Formic acid
- B: 50% AcN, 30% Methanol (MeOH), 10% 2,2,2-Trifluoroethanol (TFE), 10% Water, 0.1% Formic acid (FA)
- Autosampler loading solvent for sample injection was 0.1% aqueous TFA
- Wash of the injection needle, sample injection valve, and the trap column was performed using 100% TFE.

Trapping column used for sample loading, concentration and clean-up was a C18 PepMap, 3µm particle size, 300µm internal diameter and 5 mm length (ThermoFisher, Vienna, Austria). Nano chromatographic separation of peptides was performed on a C18 µPAC (µ-Pillar-Arrayed-Column, PharmaFluidics, Gent, Belgium). The pillars had an interpillar distance of 2µm, and the total separation path was 2µm. Both, the trap and the separation column were operated in a column oven at 45°C. Sample was loaded onto the trap column for 10 minutes when the valve switched the position and the nano gradient was directed through the trap column and onto the separation column. The trap column was switched back into the flow path of the loading column at 170 minutes in the runtime for equilibration and preparation of the following injection.

Separation was performed at 600nl/min and the gradient was formed as follows: An isocratic start with 2% B was maintained for 10 minutes and was followed by increasing the amount of B to 60% until 150 minutes. The column and the trap column were flushed with 90% B for 15 minutes, until 160 minutes, which was followed by equilibration of 25 minutes for the separation column. Blank samples (injection of loading solvent) were run between sample injections for cleaning the separation system and preventing carry-over.

Before mass spectrometric detection and analysis, peptides were also identified using the UV at 214 nm in a 3nl cell. Mass spectrometric detection and MS/MS analysis was performed using the Q-Exactive Orbitrap BioPharma (ThermoFisher, Bremen, Germany). Peptides were introduced into the nano electrospray source (ESI) after the UV cell and the ionization was performed using the steel needle with 20 µm inner diameter and 10µm tip. Needle voltage was set to 2kV in positive mode and the top 10 ions were selected for MS/MS analysis (fragmentation), resolution was set to 70.000 for full MS scans, ions with single charge were excluded from MS/MS analysis and fragmented ions were excluded for 60 seconds from further fragmentation. Raw MS/MS files were converted into “mgf” by applying *MS_Convert* (http://proteowizard.sourceforge.net/tools.shtml). Database search (Sus scrofa, http://www.uniprot.org/proteomes/UP000008227, version from November 2017) was performed by submitting the “mgf” files to MASCOT v. 2.6.0 (Matrix Science, London, UK) through ProteinScape (Bruker, Bremen, Germany) and using following parameters:

- Taxonomy: Sus scrofa
- Modifications: carbamidomethyl on C as fixed, deamidation on N and Q, carboxymethylation on M and phosphorylation S and T as variable.
- Peptide tolerance was set to 20 ppm and the MS/MS tolerance to 0.05Da
- Trypsin was selected as the enzyme used and two missed cleavages were allowed
- False discovery rate (FDR) was set to 1% and the decoy database search was used for estimating the FDR.

Fichtenbaum, A., Schmid, R., & Mitulović, G. (2016). Direct injection of HILIC fractions on the reversed-phase trap column improves protein identification rates for salivary proteins. *Electrophoresis,* 37, 29222929.


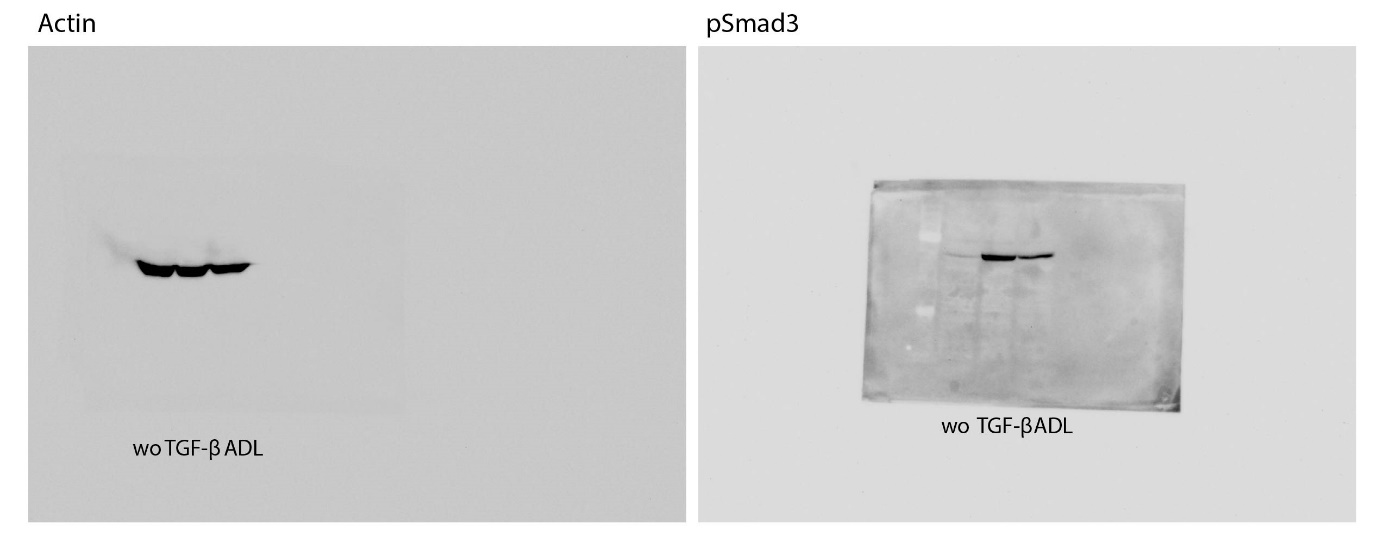


**Supplementary Figure 1.** Full-length blots, incubation of gingival fibroblasts with ADL caused an increased phosphorylation of Smad3.

**
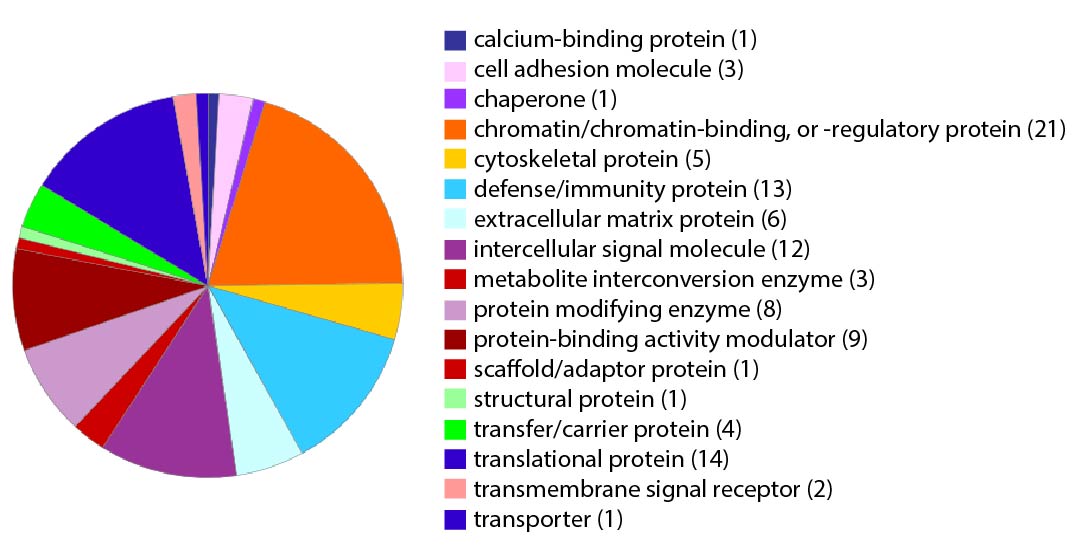
**

**Supplementary Figure 2.** Protein classes detected in porcine dentin. Numbers in parenthesis indicate number

of proteins in each protein class.


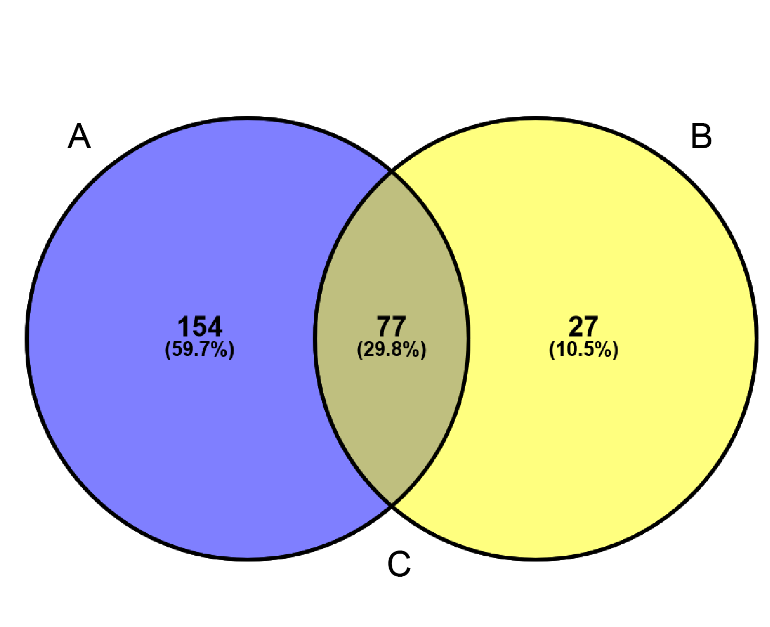


**Supplementary Figure 3.** Venny diagram. A) 154 genes regulated by ADL, independent of SB431542, B) 27 genes regulated by SB431542, independent of ADL, C) 77 SB431542-dependent genes regulated by ADL. All genes are regulated by log 2 fold change $\leq$ -3 and $\geq$ +3.

**Abbreviation Meaning**

TGF-β Transforming growth factor beta

IGF Insulin-like Growth Factor

PDGF Platelet-derived growth factor

CTGF Connective tissue growth factor

LTBP latent transforming growth factor beta binding protein 3

IGFBP IGF binding protein

COL Collagen

DCN Decorin

BGN Biglycan

LUM Lumican

FN Fibronectin

VIM Vimentin

POST Periostin

OGN Osteoglycin

NPG No Pollen Germination

MMP Matrix metallopeptidase

ENAM Enamelin

PTF-BETA Pancreas Associated Transcription Factor beta

TIMP1 Tissue Inhibitor of Metalloproteinase

CCL16 C-C motif chemokine ligand 16

CXCL12/14 C-X-C motif chemokine 12/14

LMNA Lamin A/C

NES Nestin

CHAD Chondroadherin

BMP Bone morphogenetic protein

LDLRAD Low-density lipoprotein receptor

MYOCD Myocardin

PMEPA prostate transmembrane protein androgen induced

AMTN Amelotin

SP Specificity protein

CXCR CXC-chemokine receptor

PRL Prolactin

TNFSF11 Tumor necrosis factor ligand superfamily member 11

ACTC1 α- cardiac actin 1

CD24 Cluster of differentiation 24

CRLF1 Cytokine Receptor-Like Factor 1
